# Music exposure enhances resistance to *Salmonella* infection by promoting healthy gut microbiota

**DOI:** 10.1128/spectrum.02377-24

**Published:** 2025-03-25

**Authors:** Clara Y. Zhu, Hyuntae Byun, Elyza A. Do, Yue Zhang, Ethan Tanchoco, Joris Beld, Ansel Hsiao, Jun Zhu

**Affiliations:** 1 Department of Microbiology, Perelman School of Medicine, University of Pennsylvania 332252 , Philadelphia, Pennsylvania, USA; 2 Department of Biology, Massachusetts Institute of Technology 311383 , Cambridge, Massachusetts, USA; 3 Department of Microbiology & Plant Pathology, University of California Riverside 207030, Riverside, California, USA; 4 Department of Microbiology & Immunology, College of Medicine, Drexel University 427311, Philadelphia, Pennsylvania, USA; Chengdu University, Chengdu, Sichuan, China

**Keywords:** microbiota, music therapy, Salmonella, Lactobacillus, metabolomics

## Abstract

Music intervention is gaining recognition as a cost-effective therapeutic for improving human health. Despite its growing application, the mechanisms through which music exerts beneficial health effects remain largely unexplored. Here, we show that music can exert beneficial effects in mice through modulating gut microbiome composition. Adult mice were exposed to ambient noise, Mozart’s Flute Quartet in D Major, K. 285, or white noise over a three-week period. Afterward, we observed treatment-specific changes in the community of gut commensal bacteria in these animals. Upon subsequent challenge with the bacterial pathogen *Salmonella typhimurium*, control groups exhibited significant weight loss and increased *Salmonella* colonization, whereas the Mozart-treated group did not. 16S ribosomal RNA gene sequencing revealed that the Mozart group showed a significant increase in *Lactobacillus salivarius*, a probiotic known for its antibacterial properties. Further experiments confirmed that *L. salivarius* mitigated *Salmonella* infection in mice and that *L. salivarius* acidified local environments in *in vitro* culture, thus inhibiting *Salmonella* growth. Additionally, mice exposed to Mozart consumed more food but showed similar body weight compared to the control groups. Behavioral assessments, including open field and object location tests, revealed that Mozart-treated mice were more active, less anxious, and exhibited enhanced spatial memory. Finally, Mozart exposure was shown to significantly boost colonization of administered *L. salivarius* and alter gut metabolite profiles. These findings suggest that music exposure fosters healthier gut microbiota, enhancing resistance to bacterial infections and highlighting the potential of music therapy as a novel strategy to combat drug-resistant pathogen infections.

**IMPORTANCE:**

Music therapy is increasingly recognized as a low-cost approach to improving health, but how it works remains unclear. Our study demonstrates that music can positively influence health by altering the gut microbiome. In a mouse model, exposure to Mozart’s Flute Quartet in D Major enhanced the gut microbiota, specifically increasing levels of the beneficial bacterium *Lactobacillus salivarius*. This probiotic protected mice from *Salmonella* infection by creating an acidic environment that inhibited pathogen growth. Mozart-treated mice also showed reduced anxiety, better spatial memory, and higher food intake without weight gain, suggesting the benefits of music exposure. These findings reveal a novel link between music, gut health, and disease resistance, suggesting that music therapy could be a promising strategy for enhancing gut microbiota and combating infections, including those caused by drug-resistant bacteria.

## INTRODUCTION

The gastrointestinal tract of humans and other mammals is home to a remarkably intricate community of microorganisms, collectively known as the gut microbiota (1–3). This complex ecosystem, comprising trillions of microbes, has garnered significant scientific interest due to its profound impact on host physiology. Recent advancements in research have illuminated the pivotal role of the gut microbiota and its genetic complement, the microbiome, in sustaining gut homeostasis and influencing physical and mental health. The gut microbiota is instrumental in extracting nutrients and energy from dietary intake (4), providing a defense against enteric pathogens (5, 6), and facilitating the development and maintenance of the immune system (7). Furthermore, it plays a critical role in metabolic regulation and brain programming (8). Disruptions in the delicate equilibrium between the gut microbiota and the host have been implicated in a spectrum of diseases, including neurological disorders, obesity, and cancer (9). This growing field of study underscores the importance of understanding the gut microbiota’s multifaceted interactions with the host, which may pave the way for novel therapeutic and prophylactic strategies.

*Salmonella enterica*, a Gram-negative bacterium, is one of the leading causes of foodborne diarrheal disease in the USA and globally (10). This pathogen is ubiquitous in the human food chain and can adapt to a broad range of hosts by deploying an array of virulence factors to overcome the host’s defenses and colonization resistance from the normal gut microbiota (5, 11). In recent years, the rapid emergence of antibiotic-resistant bacteria has been occurring worldwide at an alarming rate, endangering the efficacy of antibiotics (12). Cases of antibiotic-resistant *Salmonella*, including those that are resistant to clinically important drugs, are on the rise, representing a major public health concern (13). Therefore, new treatment strategies for *Salmonella* infection and a better understanding of *Salmonella* pathogenesis in the gut are urgently needed. Since the gut microbiota has been shown to drive colonization resistance against Gram-negative pathogens including *Salmonella* (14), we aimed to evaluate whether cost-effective treatments targeting the microbiota could drive prophylactic effects against *Salmonella* infection.

A number of host and environmental factors, such as immune signals, diet, and psychological stressors, can shape the gastrointestinal microbiome composition and maintain host homeostasis (15). The difficulty in identifying and validating molecular interventions in driving microbiota structure leaves an unmet need for cost-effective and easily applicable treatments to improve host health via the microbiota. One non-pharmacological method to modify host health involves music therapy. It has been reported that listening to music can result in specific physiological changes in humans by activating specific pleasure areas in the limbic system leading to the release of neuropeptides such as dopamine, and endogenous opioids, which are known to be involved in pleasure and reward control (16, 17). A large number of studies have shown that music may have the beneficial effects of reducing blood pressure in chronic hypertension patients, reducing anxiety and pain, and enhancing immune function (18). Interestingly, some studies using the rodent as an animal model have also shown that music exposure enhances the expression of neuropeptides in the limbic system (19), and stress responses represent a link between gut microbial composition and host health; recent work suggests that stress leads to an expansion of specific gut taxa such as Lactobacilli dependent on communication via the vagus nerve that links the gut to the broader nervous system (20). However, whether music has any impact on the gut microbiome is not known. As music, particularly classical music, affects the release of neuropeptides (21), we hypothesized that classical music may positively adjust gut microbiome composition. In this study, we used a mouse model to test this hypothesis and discovered that classical music promotes the expansion of specific commensal members of a healthy gut microbiota, *Lactobacillus salivarius*. We confirm that music therapy promotes colonization of externally administered *L. salivarius* and that this bacterium mediates anti-*Salmonella* effects through acidification of local microenvironments. Taken together, our findings suggest that music therapy may affect microbiota structure as a cost-effective prophylactic strategy against infection by enteric pathogens such as *Salmonella*.

## MATERIALS AND METHODS

### Bacterial strains and culture conditions

*Salmonella enterica* serovar Typhimurium SL1344 (*STm* or *Salmonella* for short) was obtained from Dr. Sunny Shin’s laboratory at the University of Pennsylvania. *Lactobacillus salivarius* (ATCC11741) and *Lactobacillus gasseri* (ATCC33323) were purchased from the American Type Culture Collection (ATCC). *Salmonella* was propagated in LB (Luria-Bertani) medium with 100 µg/mL streptomycin at 37°C. Lactobacilli were grown in MRS (de Man, Rogosa, and Sharpe) medium at 37°C. When a solid medium was used, 1.5% agar was included. For semi-solid medium, 0.3% agar was added. When *Salmonella* and *Lactobacillus* were co-cultured, LB-MRS mixed at 1:1 was used.

#### Sound treatment

Three groups of five 6-week-old adult CD-1 female mice (Charles River Laboratories) were used. Female mice were selected because they are generally more docile and less aggressive than males, which facilitates easier housing and handling, minimizing stress-related variables that could impact the study’s outcomes. Group 1 was the control group not exposed to additional sound, with an ambient noise level of around 63–70 dB. Group 2 was exposed daily to Mozart’s Flute Quartet in D Major [65–75 Decibels (dB)] (https://youtu.be/UVPhXOaTLDA?si=vxsP2qkzNG8VJRn0) for 12 h during the daytime with a 12-light/12-dark cycle. Group 3 was exposed to only white noise (set to 70 dB) (https://www.cjoint.com/c/MFvnzYqfFKA) for 12 h during the day.

### *Salmonella* infection

For *Salmonella* infection, 10^8^*Salmonella* were orally inoculated into mice that had been fasted overnight, and 50 µL of 0.5 M NaHCO_3_ was injected to neutralize the stomach acid 20 min prior to *Salmonella* infection. Mouse fecal pellets were collected and homogenized in saline using a BeadBeater. *Salmonella* colonization (colony-forming units, CFU) was determined by serially diluting the homogenates and plating on LB agar plates containing streptomycin (100 µg/mL).

### *L. salivarius* colonization

For *L. salivarius* colonization, mice were first treated with streptomycin (1 mg/mL) in their drinking water for 3 days, which continued during *L. salivarius* inoculation. The *L. salivarius* used was naturally resistant to streptomycin. Mice were inoculated intragastrically with 10^9^ CFU/mouse *L*. *salivarius* daily for 3 days. To quantify *L. salivarius* colonization, homogenized mouse fecal pellets were serially diluted and spread on MRS agar plates containing 100 µg/mL streptomycin.

### Mouse behavior tests

The experimental device used was a rectangular open field (20 × 15 in) surrounded by walls. All these elements were black. The video files were processed by a MATLAB script, MouseActivity (22). (i) Open Field Test (OFT). The procedure used is described in reference (22) with modifications. Mice were tested individually, each released from the same corner. Each testing session lasted 15 min. The arena was cleaned with 70% ethanol after each test. Thigmotaxis (i.e., the proportion of total time spent on the outer edge of the field) and distance traveled were calculated by the script.(ii) Object Location Task (OLT). The OLT test procedure used is described in reference (23) with modifications. Mice were briefly acclimated to the testing room for 30 min prior to testing. The training trial was then performed to allow mice to investigate the arena and two taped objects five inches away from two non-release corners for 10 min. The mice were then placed back in a clean holding cage (a delay called the inter-trial interval, or ITI) for 20 min before being placed in the same arena for another 10 min and then placed in their holding cages for an ITI of 20 min. For the OLT, one of the objects from the training trial was moved five inches away from another non-release corner, and the mice were allowed to explore for 10 min. The arena and the objects were wiped with 70% ethanol between each trial to minimize olfactory cues. Areas 1.5 cm around each object were defined, and the MouseActivity script was used to determine how much time mice spent exploring each object. New Object Exploration was defined as the percentage of the time a mouse spent on the object moved to another location to the total time spent on all the objects.

### Gut microbiome 16S sequencing

Prior to sound exposure (T0) and 3 weeks after sound exposure but prior to *Salmonella* infection (T1), fecal pellets were collected and physically disrupted by bead-beating, with phenol-chloroform treatment for DNA extraction (24). The V4 variable region of the 16S ribosomal RNA gene was amplified using the 515F (GTG**C**CAGCMGCCGCGGTAA) and 806R (GGACTAC**H**VGGGTWTCTAAT) primers as previously described (25). The resulting fecal amplicon libraries were then sequenced using the Illumina MiSeq platform with a read length of 2 × 150 bp, and data were analyzed using QIIME2-2021.4 (26) and VEGAN (27).

### *In vitro Salmonella-Lactobacillus* interactions

*Lactobacillus* strains were cultured in MRS medium and incubated at 37°C stationarily. *Salmonella* was cultured in LB medium at 37°C with shaking. When *Lactobacillus* and *Salmonella* were co-cultured, MRS and LB were mixed at a 1:1 ratio. A pH meter was used to measure the medium’s pH, and 0.1% bromocresol purple was used in a semi-solid medium as a pH indicator.

### Metabolite analysis

Fresh mouse fecal pellets (200 ± 10 mg) were extracted by bead beating using methanol. The solvents were evaporated, and samples were dissolved in 40 µL of 50/50 (vol/vol) water/acetonitrile. For short-chain fatty acid (SCFA) analysis, the samples were pre-column derivatized with EDC/Hobt/aniline using an established protocol (28). Authentic standards were treated the same way and were run at the same time. Samples were analyzed on a Waters Acquity I-class UPLC system coupled to a Waters Synapt G2Si HDMS mass QTOF spectrometer operated in positive ion mode utilizing a heated electrospray ionization (ESI) source. Separation was performed on a Waters Acquity UPLC BEH C_18_ 1.7 µm 2.1 × 50 mm column using a 0.6 mL/min gradient of 95/5 to 15/85 A/B in 4 min. Eluent A was 0.1% (vol/vol) formic acid in water, and B was 0.1% (vol/vol) formic acid in acetonitrile. Data were collected in MSe mode, and masses were extracted from the TOF MS TICs using a 0.005 Da abs width. Beta-diversity of overall metabolite profiles was analyzed using VEGAN (27).

### Statistical analysis

All statistical analyses were performed using GraphPad Prism software (version 10). Data are presented as mean ± standard deviation (SD) unless otherwise specified. Statistical significance was defined as *P* < 0.05. Graphs represented individual data points, means, error bars for SD/SEM. Sample sizes (*n*) and specific statistical tests for each analysis were detailed in the corresponding figure legends.

## RESULTS

### Music treatment alters gut microbiome composition

**Fig 1:**
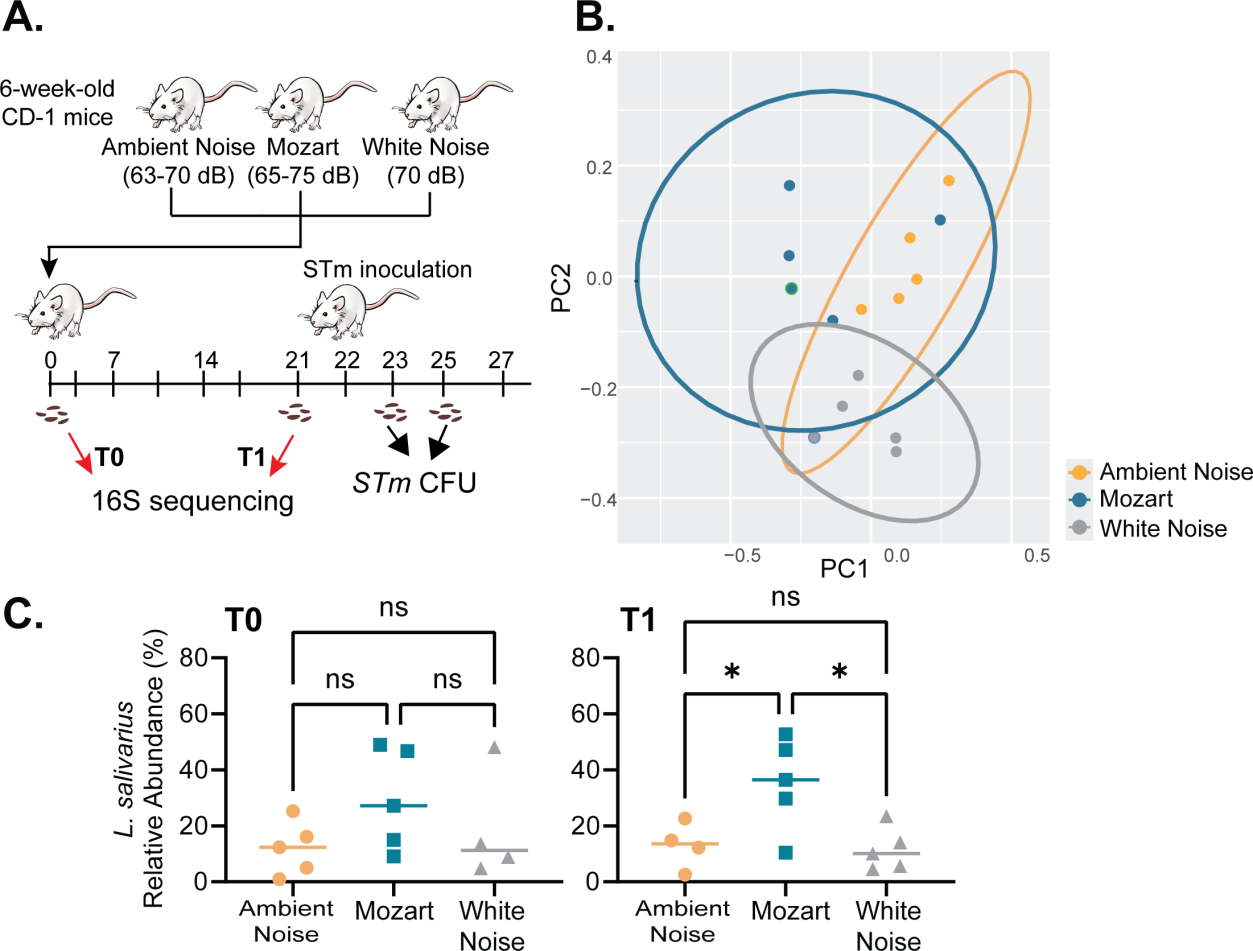
Sound treatments impact the composition of mouse gut microbiota. (A) Schematic diagram of procedures for animal experiments. T0: prior to the sound exposure; T1: 3 weeks after sound exposure. (B) Unweighted unifrac principal coordinates analysis (PCoA) of 16S gene V4 region amplicon sequencing. Percent variance is explained by each axis in parentheses. (C) Sound effects on the relative abundance of *L. salivarius*. ns, no significance; *P < 0.05 (one-way ANOVA).

To determine the effects of music on the gut microbiome, we used a mouse model that has been previously used for studying music interventions; music has been shown to elicit similar effects in rodents and humans (29, 30). Most importantly, we can closely control the diet and growth environment so that there are fewer unknown variables affecting the gut microbiome. One group of 6-week-old CD-1 mice received only ambient noise (AN), while another group of mice was exposed to Mozart’s Flute Quartet in D Major for 12 h daily at 65–75 decibels (dB). To compare the possible effects of music’s melody, rhythm, and frequency, we also exposed another group of mice to white noise (“WN,” solely as a form of auditory enrichment) at a decibel level similar to the average level of Mozart music exposure. The schematic diagram of the experimental procedures is shown in Fig. 1A. To study the impacts of music exposure on mouse gut microbiome composition, we collected mouse fecal pellets from these three groups prior to sound exposure (T0), as well as 3 weeks after sound treatment but prior to *Salmonella* infection (T1). Total DNA was extracted, and the V4 region of the microbial 16S ribosomal RNA gene was amplified and sequenced using the Illumina MiSeq platform. The resulting amplicon reads were then analyzed using the QIIME2 software pipeline (https://doi.org/10.1038/s41587-019-0209-9). We observed that the overall community structure of the fecal microbiota clustered based on sound treatment at 3 weeks (Fig. 1B). More strikingly, this analysis showed that species of Lactobacillus exhibited dramatic swings in relative abundance depending on the type of sound treatment (Fig. 1C). In particular, *L. salivarius* approached 60% relative abundance in some animals given Mozart treatment but remained near 10% in AN and WN groups. We did not observe any non-Lactobacillus species >1% relative abundance that exhibited sound-dependent differences at 3 weeks post-introduction of sound treatment. The full species-level analysis is listed in Table S1. Taken together suggesting that classical music has profound impacts on mouse gut microbiome composition, with a specific effect on *Lactobacillus*.

### Music exposure enhances resistance to *Salmonella* infection

**Fig 2:**
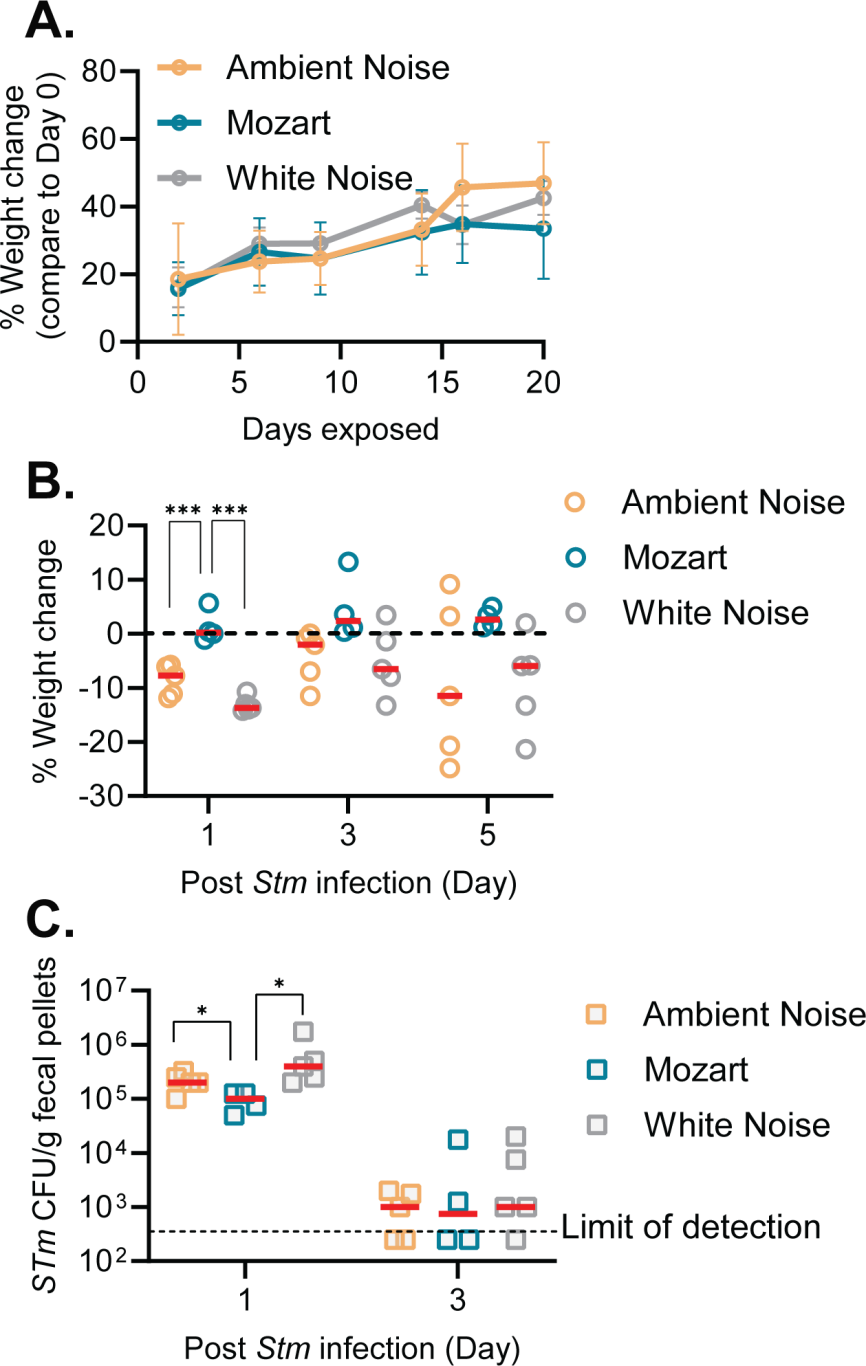
*Salmonella* infection of sound-exposed mice. (A, B) Weight changes of mice prior (A) and after (B) exposure to 10^8^/mouse *Salmonella* were orally inoculated into mice exposed to different sound treatments. In A, weight change of the weight on Day 0 of the experiment, and in B, % change of weight on Day 0 of *Salmonella* infection. ***P < 0.005 (Unpaired *t*-test). (C) Colonization of *Salmonella*. Fecal pellets were collected, and viable CFU of Salmonella was determined by serial dilution and spreading them on selective LB agar plates. *P < 0.05 (unpaired *t*-test).

During the sound treatment period, mice in all three groups gradually gained weight, and there was no statistically significant difference between the groups (Fig. 2A), suggesting that different sound exposures did not have dramatic differences in baselines host health.

To determine whether Mozart-exposed mice may have increased resistance to pathogen infection, *Salmonella* was intragastrically inoculated into mice after 3 weeks of music exposure. One day after *Salmonella* infection, mice in AN and WN groups lost ~10% of their body weight, which is characteristic of normal *Salmonella* infection in mice. The difference was statistically significant on Day 1 post-infection but disappeared on Day 3 and Day 5 (Fig. 2B). In contrast, mice in the Mozart group did not exhibit significant weight loss post infection (*P* > 0.05) by the paired *t* test of the body weight before and post infection. We also found that the number of *Salmonella* colonized in the Mozart group mice 1 day after infection was lower than that of the AN and WN groups (Fig. 2C), which may explain why Mozart mice displayed less weight loss. On Day 3, *Salmonella* colonization was reduced in all three groups (Fig. 2C). Since CD-1 mice are not as susceptible to *Salmonella* infection as other mouse strains, such as C57Bl/6J mice, *Salmonella* infection in CD-1 does not result in systematic infection (31). These data suggest that music has positive impacts on mice and may help them fight against bacterial infection. *L. salivarius* strains are well-established probiotics with multiple applications in animal health, particularly to reduce the colonization of gastrointestinal pathogens (32). These data imply that the resilience of mice in the Mozart group to *Salmonella* infection (Fig. 2B) may be the result of the increased presence of *L. salivarius* (Fig. 1C) and that exposure to Mozart may result in the bloom of this *Lactobacillus* species.

### *L. salivarius* inhibits *Salmonella* colonization and *in vitro* growth by producing acids

**Fig 3:**
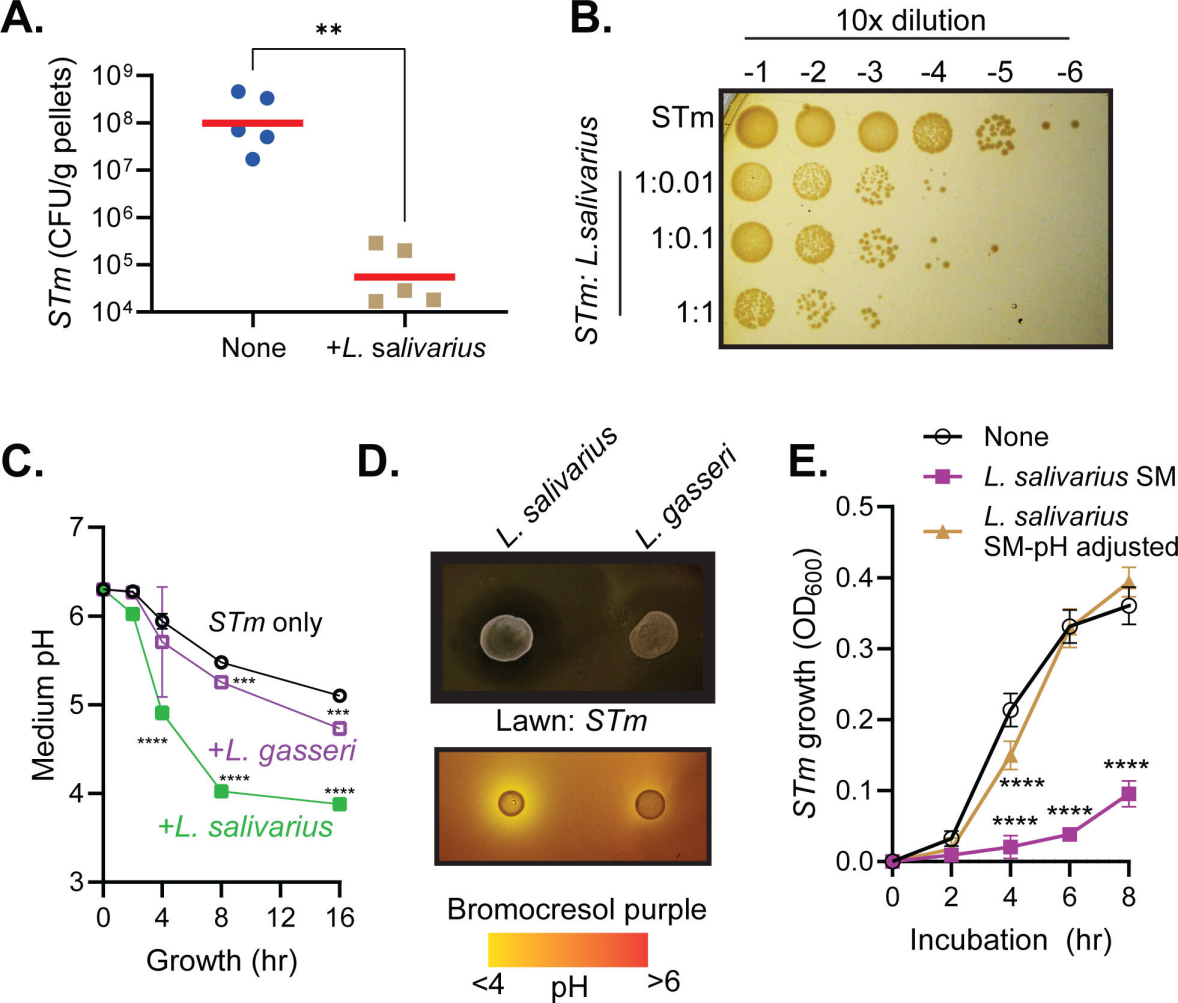
*L. salivarius* inhibits *Salmonella* growth *in vivo* and *in vitro*. (A) *L. salivarius* inhibits *Salmonella* growth *in vivo*. Six-week-old female CD-1 mice were given drinking water containing 1 mg/mL streptomycin for 1 day. PBS buffer (empty circles) or 10^9^ CFU/mouse *L*. *salivarius* (green squares) were then intragastrically inoculated into mice. Two days later, 10^8^ CFU/mouse *Salmonella* were orally inoculated into mice. Fecal pellets were collected after 1 day of infection and colonized *Salmonella* were enumerated. Horizontal lines: Geometric mean of 5 mice. **P < 0.01 (*t*-test). (B) *Salmonella* survival rate in the presence of *L. salivarius*. 10^6^ CFU/mL of *Salmonella* and different amounts of *L. salivarius* were inoculated into MRS/LB (50:50) media and incubated at 37°C without shaking for 16 h. The cultures were then serially diluted and spotted onto LB agar plates. The plates were then incubated at 37°C for 16 h before being photographed. *L. salivarius* was unable to grow on these plates. (C) 10^7^/mL *Salmonella* without or with 10^7^/ml *L*. *gasseri and L. salivarius* were inoculated into MRS/LB and incubated at 37°C stationarily. Medium pH was measured by a pH meter at the time point indicated. *N* = 3. ****P < 0.0001 (*t*-test) (compared to the medium pH of *Salmonella* only at the corresponding time points). (D) *Salmonella* was seeded in the MRS/LB media containing 0.3% agar without (top) and with (bottom) the pH indicator bromocresol purple (0.1%). Ten microliters of overnight cultures of *L. salivarius* and *L. gasseri* was then spotted. The plates were incubated at 37°C for 16 h before photographed. (E) The effects of acidified media on *Salmonella* growth. 10^7^/mL *Salmonella* were inoculated into LB containing 50% fresh MRS, *L. salivarius*-grown spent media (SM), and spent media adjusted to pH 6.5 using NaOH. The cultures were incubated at 37°C stationarily. OD_600_ was measured. *N* = 12. ****P < 0.0001 (*t*-test) (compared to OD_600_ of fresh MRS at the corresponding time points).

To further study if music induces the increased presence of *L. salivarius* in the mouse gut, which may contribute to *Salmonella* resistance, we first tested whether *L. salivarius* could reduce *Salmonella* colonization independent of sound treatment. After feeding mice with streptomycin in drinking water for 3 days to deplete endogenous commensal microbes, PBS (phosphate-buffered saline) buffer or *L. salivarius* was first inoculated into mice. *Salmonella* was then inoculated into these mice 2 days later. Strikingly, we found that *Salmonella* colonization was greatly reduced in the mice that had been pre-colonized with *L. salivarius* (Fig. 3A), suggesting that *L. salivarius* can prevent *Salmonella* infection in the mouse gut. These data also indicate that the above findings, in which Mozart-exposed mice were more resistant to *Salmonella* (Fig. 2B and C), may be due to the increase of *L. salivarius* in their guts.

To determine the possible mechanism of *L. salivarius* inhibition of *Salmonella* infection, we co-cultured *Salmonella* with *L. salivarius in vitro. L. salivarius* could not grow in LB medium, and *Salmonella* could not in MRS medium, but both strains grew well in a 50% LB and 50% MRS mix. We, therefore, used this culture condition for all the *in vitro* co-culture experiments below. Figure 3B shows that *L. salivarius* effectively inhibited the growth of *Salmonella* compared to when 10^6^/mL *Salmonella* grew alone, as incubating 10^6^/mL *Salmonella* with only 10^4^/mL *L*. *salivarius* for 16 h reduced *Salmonella* growth by approximately 100-fold. The more *L. salivarius* that was included, the stronger the inhibition it displayed (Fig. 3B). These results suggest that *L. salivarius* can effectively inhibit *Salmonella* growth *in vitro*.

Because it is known that *Salmonella* is sensitive to low-pH environments (33) and that *Lactobacilli* produce lactic acid as the major metabolic end-product of carbohydrate fermentation (34), we hypothesized that *L. salivarius*’s inhibition of *Salmonella* may be due to its ability to acidify the medium rapidly. To monitor pH changes, we grew *Salmonella* alone or with *Lactobacilli* in a liquid medium. We found that in the presence of *L. salivarius*, the medium pH dropped rapidly and reached a pH of 4 in 16 h. In contrast, when *Salmonella* was grown alone or co-cultured with a different *Lactobacillus* strain, *L. gasseri*, the pH of the culture media reduced gradually and remained around 5 (Fig. 3C).

To test if it is the low pH that inhibits *Salmonella* growth, we spotted *L. salivarius* culture on soft agar plates that contained either *Salmonella* (Fig. 3D, top panel) or the pH indicator bromocresol purple (Fig. 3D, lower panel). We found a clear zone around the *L. salivarius* culture, which corresponded to the lower pH in the medium, suggesting that *L. salivarius* inhibits *Salmonella* growth through medium acidification. *L. gasseri*, on the other hand, had little effect on medium pH and *Salmonella* growth (Fig. 3D). To investigate further whether medium acidification was the main reason that *L. salivarius* inhibits *Salmonella* growth, we tested *Salmonella* growth in the cell-free spent medium of *L. salivarius* (pH ~4) and the same spent medium with the pH adjusted to 6.5. Figure 3E shows that the *L. salivarius* spent medium greatly inhibited *Salmonella* growth. The inhibitory activity was mostly diminished when the spent medium was adjusted to a pH of 6.5 prior to adding to the *Salmonella* culture (Fig. 3E, beige line), suggesting that *L. salivarius*-induced medium acidification may be the major mechanism of its inhibition of *Salmonella* growth, at least *in vitro*. Noticeably, at the 4 h time point, the pH-adjusted spent medium still had a slight but statistically significant inhibition of *Salmonella* growth (Fig. 3E). This suggests that other inhibitory mechanisms may be involved. Taken together, these data suggest that *L. salivarius* may be the reason why *Salmonella* infection was contained in the music-exposed group of mice.

### Music promotes *L. salivarius* colonization

**Fig 4:**
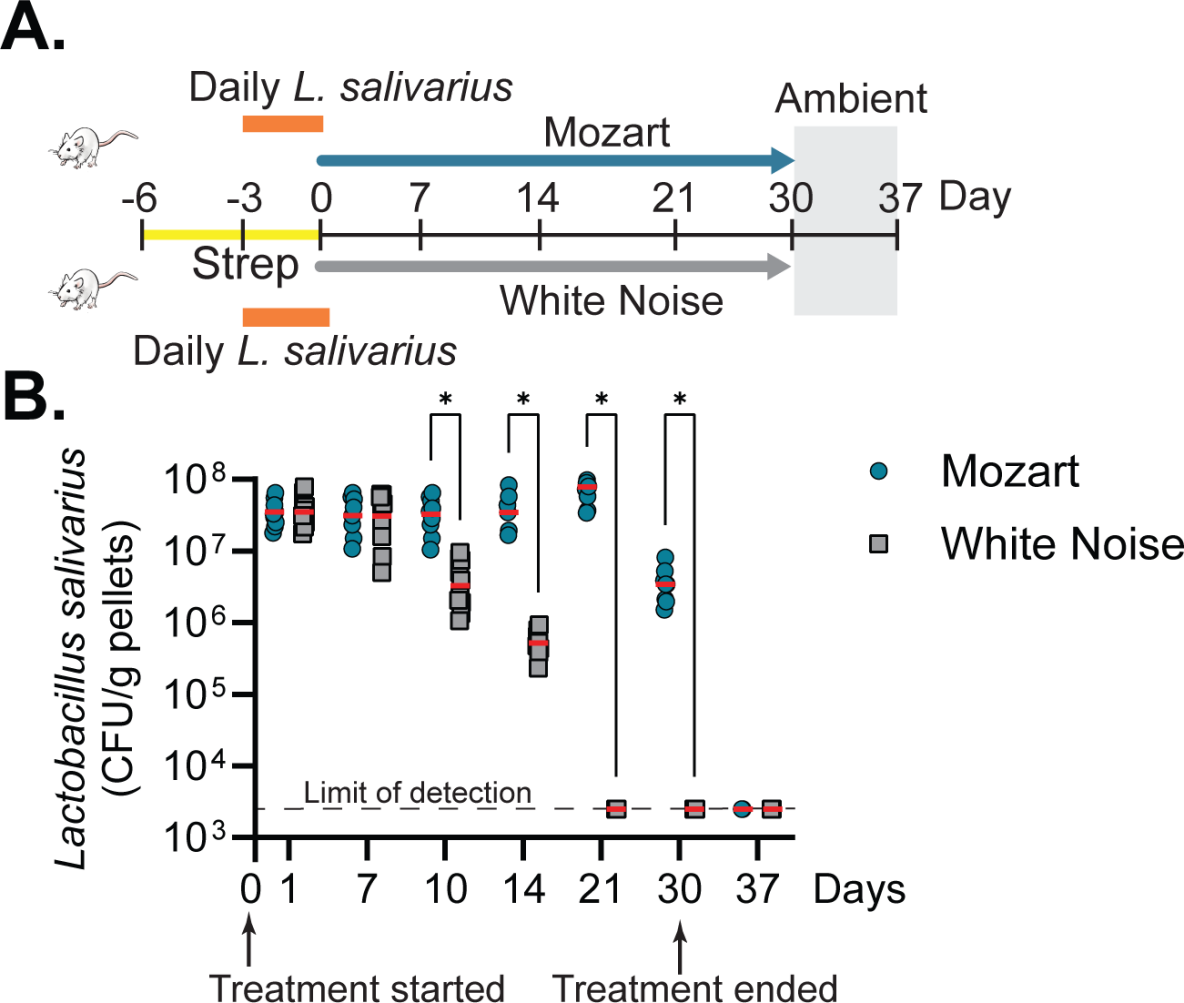
The sound effects on *L. salivarius* gut colonization. (A) Schematic diagram of procedures for animal experiments. Six-week-old CD-1 mice were treated with streptomycin (1 mg/mL) in their drinking water for 3 days and continued during *L. salivarius* inoculation. The *L. salivarius* used was naturally resistant to streptomycin. Mice were inoculated intragastrically with 10^9^ CFU/mouse *L*. *salivarius* daily for 3 days. Mice were then exposed to the music or white noise, as described in the Materials and Methods. From days 30 to 37, the sound treatment was removed. (B) *L. salivarius* colonization. At the time points indicated, fecal pellets were collected and *L. salivarius* colonization was determined by serial diluting the fecal homogenates and plating on the MRS plates containing 100 µg/mL streptomycin. *N* = 5. *P < 0.05 (unpaired *t*-test).

To confirm that music may promote *L. salivarius* colonization, we first administered streptomycin to mice to reduce their normal gut flora. We then orally inoculated 10^9^ CFU/mouse *L*. *salivarius* daily for 3 days until stable colonization was established. One group of mice was then exposed to Mozart’s Flute Quartet in D Major, while the other group was exposed to white noise. On day 30, the sound treatments were discontinued. We assessed *L. salivarius* colonization by serially diluting fecal pellets and plating on selective media. Initially, *L. salivarius* colonization levels were similar between both groups (Fig. 4B). However, after 10 days, *L. salivarius* levels remained high in the music-exposed mice but declined in the white-noise group. By week 3, *L. salivarius* was undetectable in the white-noise group but remained highly colonized in the music-exposed mice. Notably, 1 week after the cessation of music treatment, *L. salivarius* levels rapidly decreased in the previously music-exposed mice (Fig. 4B). These results suggest that music exposure significantly enhances *L. salivarius* colonization.

### Music exposure changes mouse behaviors

**Fig 5:**
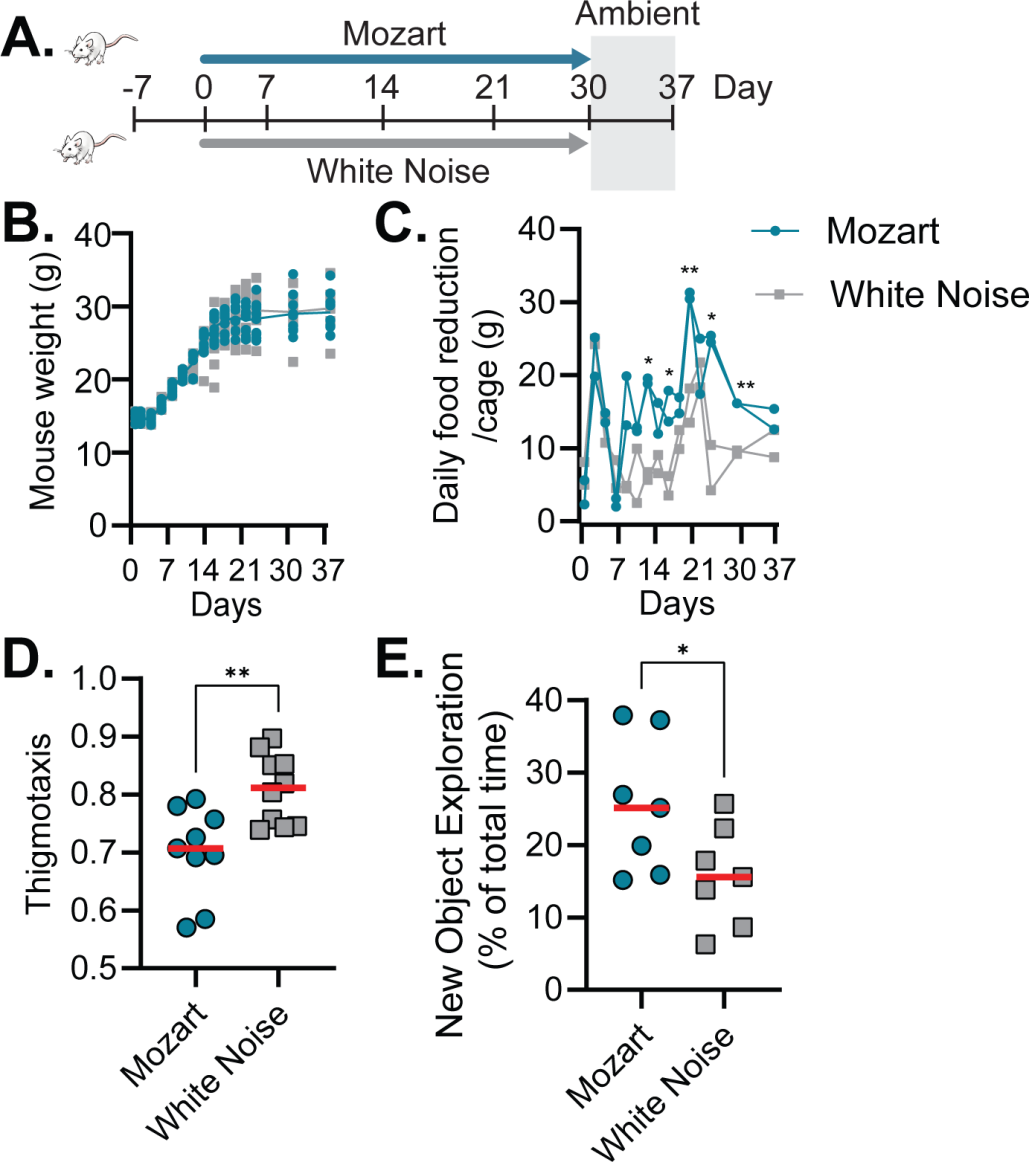
Music’s effects on mouse behavior. (A) Schematic diagram of procedures for animal experiments. After acclimatization, the sound treatment was from day 0 to day 30 and then removed from day 30 to day 37. (B) Percentage of weight change. The weight of each mouse was compared with the weight of the same mouse 1 day prior. (C) Food consumption, representing the weight of food placed in each cage (5 mice) minus the weight of remaining food after 24 h. *P < 0.05, **P < 0.005 (unpaired two-sample *t*-test for Mozart vs White Noise). (D) Open Field Tests. Thigmotaxis plot (proportion of time spent in the outer peripheral of the arena). Horizontal lines: mean of at least 9 mice. ns, no significance; **P < 0.01 (unpaired *t*-test). (E) Task New Object Exploration Task, showing the percentage of time spent on exploration of the moved object over the total time spent on exploring all the objects. Horizontal lines: mean of 7 mice. *P < 0.05 (unpaired *t*-test).

During the initial sound treatment experiment (Fig. 1A), we noticed that after 1 week of sound exposure, mice in the Mozart group were often noticeably more active compared to the other two control groups. To investigate whether music exposure may affect mouse physiology, we compared two groups of mice that were exposed to either Mozart or white noise (Fig. 5A). Although the changes in weight were similar between the two groups (Fig. 5B), we measured daily food consumption and found that the Mozart group consumed significantly more food than the white noise group (Fig. 5C). Alternatively, the Mozart mice may have “played” with their food more, which would have resulted in more food pellets breaking and falling onto the cage floor and the appearance that they consumed more food. Nevertheless, these data suggest that the mice exposed to music were more active. To quantify the effects of music on mouse behavior, we then performed an open field test, a standard approach to determine mice’s free exploratory behavior in a novel environment. When mice are introduced into a novel environment, they engage in exploration with locomotor activity mainly located along the chamber lateral walls. This tendency to remain close to the walls of an open field is called thigmotaxis and is used as an index of anxiety in mice (35). Figure 5D shows the Mozart-exposed mice had lower thigmotaxis than those of white noise-exposed, suggesting that Mozart-exposed mice were more exploratory, tending to be in the center more often, and, thus, less anxious. In addition, we performed an object location task that examined mice’s spatial memory (23). In general, mice spend more time exploring novel objects, so if they recognize the relocation of an object, they will spend more time investigating the moved object. Mozart-exposed mice were shown to have better spatial memories, as they spent more time exploring the new object (Fig. 5E). Taken together, these data suggest that Mozart-exposed mice are more active, less anxious, and have improved spatial memories compared to the control mice.

### Music exposure alters the landscape of gut metabolites

**Fig 6:**
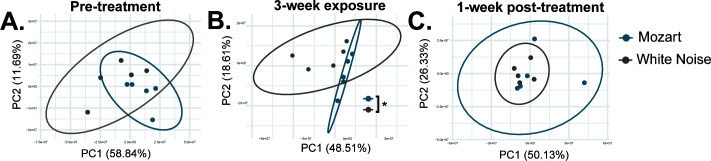
The sound effects on host metabolomics. The procedures for animal experiments were the same as in Fig. 5A. Fecal samples were collected prior to the sound treatment (A), 3 weeks after the treatment (B), and 1 week after the treatment stopped (C). PCoA of Euclidean distances between metabolite peak area across samples showed a clear separation and grouping of Mozart and WN samples, suggesting a clear difference in metabolites and metabolite abundance between these groups. *P < 0.05 (PERMANOVA).

To investigate whether music treatment could significantly influence mouse gut microenvironments and physiology, and to uncover potential mechanisms by which music exposure might enhance *L. salivarius* colonization, we performed metabolomic analysis. The procedures for animal experiments were the same as described in Fig. 5A. Fecal pellets from mice under different treatments and at different time points were extracted using methanol. The methanol-extracted samples underwent analysis via liquid chromatography-mass spectrometry (LC-MS), and the data were processed using the XCMS server in a pairwise comparative manner. Principal coordinates analysis (PCoA) revealed a clear separation and distinct groupings between the Mozart and white noise samples 3 weeks post-treatment (Fig. 6B) though this distinction was not observed 1 week after the discontinuation of treatment (Fig. 6A and C). This finding indicates a significant difference in both the types and abundances of metabolites between the two groups. The observed changes in metabolite profiles suggest that music might influence gut physiology by altering microbial activity or composition, or host metabolism.

## DISCUSSION

Extensive research has demonstrated that non-invasive, cost-effective music therapy provides significant positive benefits for patients in clinical settings (36). These benefits include reduction of stress and blood pressure, strengthening of the immune system, and improved pain management. However, the impact of music on gut microbiome composition and pathogen resistance has never been elucidated. In this study, we used a mouse model to investigate the effects of classical music on modulating gut microbiota and *Salmonella* infection. We found that the gut microbiomes were profoundly altered by music exposure, which included -exposed mice harboring more probiotic-like bacteria such as *L. salivarius*; this led to the reduction of *Salmonella* colonization. We also found that the Mozart mice were less anxious and had better spatial memories. A recent study (37) reported that the musical intervention caused modifications in the gut microbial composition of mice. However, our study demonstrated that music plays an important role in the improvement of health outcomes through the manipulation of the gut microbiome’s composition and concomitant anti-pathogen activity.

Microbial antibiotic resistance is a major public health concern. The commercialization of new antibiotics has also dramatically slowed in recent years due to decreased profitability, which raises the dangerous prospect of increasingly limited effective therapeutic options against bacterial diseases (38). Therefore, there is an urgent need for new antibacterial strategies to reduce antibiotic use and prevent the rise of antibiotic resistance. One strategy is to expand the usage of probiotics to treat infectious diseases (39, 40). When consumed or applied to the body, probiotics provide health benefits such as inhibiting pathogen adhesion, enhancing mucosal barrier function, and strengthening the immune system. But one limit of probiotic treatments is the diminished viability of orally administrated probiotics by host antimicrobial activities (such as gastric acid and bile) and competition from commensal bacteria (41). Our study found that music could increase the abundance of *L. salivarius* among gut microbiota (Fig. 1C) and prolong its colonization (Fig. 4B). These findings may have practical implications for the improved effectiveness of probiotic treatment that may contribute to the reduction of antibiotic resistance. Because music therapy is a highly cost-effective form of treatment compared to antibiotics or probiotics, therapeutic or prophylactic interventions based on music may also help to address issues of health equity.

In this study, we exposed mice to Mozart’s Flute Quartet in D Major. A study in 1993 reported that listening to a 10 min session of Mozart’s Piano Sonata, K. 448 improved college students’ performance in spatial reasoning (42). The results from this study were greatly exaggerated in the popular press, and the idea that listening to classical music can enhance one’s intelligence became known as the “Mozart effect.” The results from the original study are controversial: some investigators have been unable to reproduce the findings, but others have confirmed that listening to Mozart K. 448 produces a small increase in spatial-temporal performance (43–46). Many researchers agree that any effects that Mozart may have are due to “enjoyment arousal,” (47) but this interpretation is countered by several animal experiments (48). For future directions, it would be interesting to test other types of classical music (e.g., Romantic, Baroque, Modern), as well as different genres of music to determine whether this pathogen resistance effect is specifically induced by music composed by Mozart. Further studies into what exact component of music creates this effect could also be conducted, testing characteristics such as frequency, tempo, and rhythm. Additionally, because female mice were used in all the experiments we performed above, male mice should be also tested in the future, as it has been reported that certain effects of music may depend on an ovarian steroid in female mice (49).

Why Mozart-exposed mice are prone to have increased levels of certain *Lactobacilli* in the gut microbiota is not clear. In recent years, increasing lines of evidence demonstrate that gut microbiota play important roles in host neurodegenerative disorders, such as Alzheimer’s Disease (50) and mental health (51), but the manner in which the host’s neurological activities impact the gut microbiome is less understood. It has been shown that listening to music activates specific pleasure areas in the limbic system, which, in turn, leads to the release of neuropeptides (52). Neuropeptides are important mediators both within the nervous system and between the nervous system and other parts of the body, likely playing a role in bidirectional gut-brain communication (53). Therefore, they may influence the activity of gastrointestinal microbiota and their interactions with the central nervous system through the gut-brain axis, creating a link between music exposure and changes in the gut microbiome. In this study, Mozart-exposed mice were more active and less anxious (Fig. 5), suggestive of changes in neuropeptide production; their gut metabolite landscapes were also altered (Fig. 6). These changes may directly or indirectly improve the survivability of *L. salivarius* in their guts. A recent study (20) discovered that Brunner’s glands in the small intestine play a role in connecting stress responses in the brain with the balance of bacteria in the gut. When the vagus nerve was stimulated, these glands helped boost the levels of *Lactobacillus* in the gut. In the future, more studies will be needed to investigate whether music exposure may act through these same pathways and to determine if there are other health benefits associated with music-enhancement of specific probiotic or commensal bacteria.

## SUPPLEMENTAL MATERIAL

Table S1 spectrum.02377-24-s0001.xlsx Full species-level analysis.

ASM does not own the copyrights to Supplemental Material that may be linked to, or accessed through, an article. The authors have granted ASM a non-exclusive, world-wide license to publish the Supplemental Material files. Please contact the corresponding author directly for reuse.
